# Three-year real-world effectiveness, treatment persistence, and planned discontinuation of anti-calcitonin gene-related peptide monoclonal antibodies for migraine prevention: a single-center cohort from Japan

**DOI:** 10.3389/fneur.2026.1827022

**Published:** 2026-05-13

**Authors:** Hideyo Kasai, Taro Yasumoto, Shota Kosuge, Ayako Osanai, Keita Mizuma, Akinori Futamura, Takeshi Kuroda, Kenjiro Ono, Hidetomo Murakami

**Affiliations:** 1 Department of Neurology, Showa Medical University School of Medicine, Tokyo, Japan; 2 Department of Neurology, Showa Medical University Fujigaoka Hospital, Yokohama, Kanagawa, Japan; 3 Department of Neurology and Neurobiology of Aging, Graduate School of Medical Sciences, Kanazawa University, Kanazawa, Ishikawa, Japan

**Keywords:** 36-month follow-up, calcitonin gene-related peptide, Japan, migraine, monoclonal antibodies, monthly migraine days, real-world evidence, treatment persistence

## Abstract

**Introduction:**

Long-term real-world evidence beyond 24 months for anti-calcitonin gene-related peptide (CGRP) monoclonal antibodies is scarce, particularly regarding treatment persistence and planned discontinuation after goal attainment.

**Methods:**

We conducted a single-center retrospective study of consecutive patients aged ≥15 years who initiated galcanezumab or fremanezumab (monthly/quarterly) between May 2021 and June 2022. Specifically, patients aged ≥15 years with episodic migraine (EM; <15 headache days/month), high-frequency EM (HFEM; 8–14 days/month), or chronic migraine (CM; ≥15 days/month) were included according to the International Classification of Headache Disorders, 3rd edition (ICHD-3) criteria. The outcomes included monthly migraine days (MMDs), the Migraine Disability Assessment Scale (MIDAS), the Headache Impact Test (HIT-6), and the Visual Analogue Scale (VAS), which were assessed at baseline and at 1, 3, 6, 12, and 36 months. Responder rates (RRs) (≥50%, ≥75, and 100%) and continuation/discontinuation reasons were summarized with prespecified and sensitivity analyses. The primary subgroup analysis was prespecified as EM vs. HFEM+CM.

**Results:**

Overall, 50 patients were analyzed at baseline (mean age: 42.5 years; 88% women); 28 of the 50 (56%) patients continued therapy for 3 years. Among patients who continued therapy, MMDs decreased from 12.0 ± 5.4 to 5.6 ± 5.4 at 36 months, with parallel improvements in the MIDAS, HIT-6, and VAS (all *p* < 0.01). Responder rates were durable (≥50%: 55.6% at 36 months; ≥75%: 29.6%; 100%: 11.1%). Discontinuation frequently reflected treatment completion after goal attainment (24%); no adverse-event–related discontinuations occurred.

**Conclusion:**

Over 3 years, anti-CGRP monoclonal antibodies provided sustained preventive effectiveness and favorable tolerability in routine practice, supporting individualized decision-making regarding continuation, planned discontinuation, and regimen selection.

## 1 Introduction

Migraine causes recurrent, disabling headaches that impair work productivity, social functioning, and quality of life. While acute therapies such as triptans and lasmiditan are effective for many patients, a significant subset continues to experience frequent attacks, medication overuse headache (MOH), or persistent disability despite optimized acute care, thereby prompting the need for preventive treatment. Anti-calcitonin gene-related peptide (CGRP) monoclonal antibodies have reshaped migraine prevention by targeting a key neuropeptide involved in migraine pathophysiology (1–6). Randomized trials consistently demonstrate reductions in monthly migraine days (MMDs) and improvements in patient-reported outcomes (PROs) (1–6). Furthermore, early real-world studies corroborate these benefits in routine practice (7–9).

However, long-term real-world evidence (RWE) beyond 12–24 months remains limited, particularly regarding treatment persistence, reasons for discontinuation, and sustained effects on disability and daily impact. These data are crucial for informing expectations in clinical care, guiding shared decision-making on treatment continuation or planned cessation, and identifying patient subgroups that might benefit from individualized dosing strategies.

We conducted a single-center, retrospective analysis of consecutive patients who initiated anti-CGRP therapy (galcanezumab or fremanezumab) and were followed for up to 3 years in routine practice. Our aims were to (i) quantify long-term changes in MMDs and PROs (the Migraine Disability Assessment Scale [MIDAS], the Headache Impact Test [HIT-6], and the Visual Analogue Scale [VAS]); (ii) describe responder rates (RRs) (≥50%, ≥75, and 100% reduction in MMDs) and treatment persistence; and (iii) summarize real-world reasons for discontinuation, including treatment completion after goal attainment. By focusing on outcomes of direct clinical relevance and durability of benefit, this study adds practical, longitudinal evidence to support informed preventive care.

## 2 Materials and methods

### 2.1 Study design and setting

We performed a retrospective observational study at a tertiary neurology/headache clinic. Consecutive patients with migraine who initiated anti-CGRP monoclonal antibody therapy between 1 May 2021 and 30 June 2022 were identified and analyzed. The study adhered to the Strengthening the Reporting of Observational Studies in Epidemiology (STROBE) guidelines and was approved by the institutional ethics committee (Showa Medical University School of Medicine; approval No. 3476; 22 June 2021). During the study period in Japan, eptinezumab was not approved and was therefore unavailable for routine clinical use. Although erenumab received national marketing approval in June 2021, our university hospital formulary restricted the introduction of multiple agents within the same therapeutic class; consequently, erenumab was not adopted locally during the index period. The real-world cohort therefore comprised patients initiating galcanezumab or fremanezumab.

### 2.2 RWE design and reporting

This retrospective cohort was designed and reported in line with the International Headache Society guideline for real-world evidence studies in migraine and was written in accordance with the STROBE statement; the completed checklist is provided in Supplementary Table S1 (10, 11). Key RWE considerations included (i) prespecification of outcomes and time windows in routine care; (ii) transparent description of data provenance (clinic diaries/smartphone applications verified at visits); (iii) handling of missingness and attrition; and (iv) sensitivity analyses to assess robustness under plausible sources of bias (selection, confounding, and information bias) (10, 11).

### 2.3 Participants

The inclusion criteria were patients with a clinical diagnosis of migraine according to the International Classification of Headache Disorders, 3rd edition (ICHD-3) and who underwent treatment with galcanezumab or fremanezumab based on national guidance for appropriate preventive use. The exclusion criteria were patients with headache disorders other than migraine (except tension-type headache) or those deemed ineligible by the investigator. Of the 50 patients analyzed at baseline, 28 continued therapy for 3 years and were included in long-term effectiveness analyses.

### 2.4 Interventions

Patients received galcanezumab or fremanezumab at the physicians’ discretion, consistent with approved dosing schedules. Fremanezumab was administered either monthly or quarterly. Concomitant medications and changes in acute therapy were managed per usual care. Switching between anti-CGRP monoclonal antibodies was permitted at the physician’s discretion in routine care.

### 2.5 Outcomes and follow-ups

Assessments were performed at baseline and 1, 3, 6, 12, and 36 months. The primary endpoints were the changes in MMDs and the ≥50% RR. The secondary endpoints included changes in the MIDAS, HIT-6, and VAS; ≥75 and 100% RRs; treatment continuation; and reasons for discontinuation. Patients were instructed on diagnostic criteria and diary methods; physicians verified diary entries or smartphone application records at visits. Planned discontinuation after goal attainment was operationally defined as pausing anti-CGRP monoclonal antibody therapy after achieving a ≥50% reduction in MMDs sustained across at least two consecutive scheduled assessments (i.e., a ≥ 3-month interval; e.g., months 1 → 3 or 3 → 6), in conjunction with shared decision-making between the patient and the clinician. This definition was used to classify “treatment completion” and to distinguish it from other reasons for discontinuation (self-discontinuation, referral, or switching). Injections were administered during scheduled clinic visits; adherence to dose timing (e.g., on-schedule dosing windows) was not prospectively captured.

### 2.6 Subgroups

Chronic migraine (CM) was defined according to ICHD-3 criteria as ≥15 headache days/month for >3 months, including ≥8 migraine days. HFEM was defined as 8–14 headache days/month. Episodic migraine (EM) was defined as <15 headache days/month, with HFEM treated *a priori* as a clinically high-burden subset. In routine practice, HFEM and CM represent the principal candidates for preventive therapy owing to their greater associated disability and increased risk of chronification. Accordingly, our prespecified primary subgroup analysis contrasted EM vs. HFEM+CM to reflect real-world treatment decision-making. Baseline characteristics are additionally shown across low-frequency episodic migraine (LFEM), HFEM, and CM for transparency. Analyses were performed to summarize overall outcomes and stratified descriptively by episodic migraine (EM) and high-frequency episodic/chronic migraine (HFEM+CM) to reflect clinical burden relevant to preventive decisions. High-frequency episodic migraine (HFEM) was considered a subset of EM throughout the analyses. Consistent with ICHD-3 definitions, HFEM and CM reflect a greater attack burden and risk of progression and thus are typical candidates for preventive therapy (12). In line with this finding, the latest American Headache Society position statement recognizes CGRP-targeting therapies as a first-line option for migraine prevention without *a prior*-failure requirement, supporting early, individualized initiation of preventive care (13). Accordingly, we prespecified EM and HFEM+CM subgroups to reflect clinically significant decision points for prevention. Drug- and dosing-specific time series are presented in the Supplementary material.

### 2.7 Statistical analysis and handling of bias

We conducted prespecified sensitivity analyses to assess robustness to attrition and missing outcomes in this long-term real-world cohort. Specifically, we fitted a generalized linear mixed model (GLMM) with a Gaussian distribution and identity link, including a random intercept for patient and time as a categorical fixed effect (models were optionally adjusted for baseline values and EM vs. HFEM/CM where applicable). In addition, we performed multiple imputation (*m* = 50) for change-from-baseline values at each time point, with estimates combined using Rubin’s rules. Model-based mean changes at each time point (estimated marginal means) and corresponding two-sided 95% confidence intervals were derived from the fitted GLMM. Wald tests were used to assess the null hypothesis of no change from baseline at each time point. Full model estimates are provided in Supplementary Table S3.

## 3 Results

### 3.1 Baseline characteristics

Among the 50 patients (mean age: 42.5 ± 11.7 years; 88.0% women), EM was present in 33 (66.0%) patients, HFEM in 20 (40.0%), and CM in 17 (34.0%). Migraine without aura occurred in 37 (74.0%) patients, and migraine with aura occurred in 13 (26.0%) patients. MOH was observed in 25 (50.0%) patients. Initial treatments were galcanezumab (*n* = 24), fremanezumab monthly (*n* = 10), and fremanezumab quarterly (*n* = 16). Baseline demographics stratified by treatment regimen are summarized in Table 1. Baseline characteristics are also provided in Table 2, stratified by LFEM, HFEM, and CM, to facilitate the interpretation of subgroup burden and to ensure transparency in subgroup allocation.

**Table 1:** Patient characteristics at baseline and among 3-year continuers, by treatment regimen.

Patient group	ALL patients	Galcanezumab	Fremanezumab monthly	Fremanezumab quarterly
Number of patients	50	24	10	16
Age, years	42.5 ± 11.7	43.5 ± 11.7	46.9 ± 6.8	38.3 ± 13.3
Sex, female (%)	44 (88.0)	22 (91.7)	10 (100.0)	12 (75.0)
Patients with EM (*n*, (%))/patients with HFEM (*n*, [%])	33 (66.0)/20 (40.0)	16 (66.7)/9 (37.5)	5 (50.0)/4 (40.0)	12 (75.0)/7 (43.8)
Patients with CM (*n*, [%])	17 (34.0)	8 (33.3)	5 (50.0)	4 (25.0)
Migraine without aura (*n*, [%])	37 (74.0)	19 (79.2)	8 (80.0)	10 (62.5)
Migraine with aura (*n*, [%])	13 (26.0)	5 (20.8)	2 (20.0)	6 (37.5)
Medication overuse headache (*n*, [%])	25 (50.0)	12 (50.0)	5 (50.0)	8 (50.0)
Onset, years	22.4 ± 7.9	22.1 ± 7.5	24.5 ± 6.6	21.5 ± 9.4
Disease history, years	20.1 ± 9.8	21.4 ± 9.8	22.4 ± 8.9	16.8 ± 10.2
Disease duration from the first visit, years	4.67 ± 4.08	4.25 ± 4.00	5.42 ± 4.75	4.92 ± 3.83
Outcome				
Continuous anti-CGRP use (3 years, *n* [%])	28 (56.0)	12 (50.0)	6 (60.0)	10 (62.5)
Self-discontinuation (3 years, *n* [%])	6 (12.0)	4 (16.7)	0 (0.0)	2 (12.5)
Planned discontinuation (treatment completion) (3 years, *n* [%])	12 (24.0)	5 (20.8)	3 (30.0)	4 (25.0)
Anti-CGRP switch (3 years, *n* (%)), e.g., FRE(M) → GAL, GAL→FRE(Q)	1 (2.0), GAL→FRE(Q)	1 (4.2), GAL→FRE(Q)	0 (0.0)	0 (0.0)
Referral (3 years, *n* (%))	3 (6.0)	2 (8.3)	1 (10.0)	0 (0.0)

Continuous anti-CGRP use (3 years, *n*)	28	12	6	10
Age, years	43.5 ± 9.4	44.7 ± 8.2	45.8 ± 2.7	40.6 ± 12.8
Sex, female (%)	24 (85.7)	11 (90.0)	6 (100.0)	7 (70.0)
Patients with EM (*n*, [%])/patients with HFEM (*n*, [%])	20 (71.4)/13 (46.4)	8 (66.6)/6 (50.0)	3 (50.0)/2 (33.3)	9 (90.0)/5 (50.0)
Patients with CM (*n*, [%])	8 (28.6)	4 (33.3)	3 (50.0)	1 (10.0)
Migraine without aura (*n*, [%])	21 (75.0)	9 (75.0)	5 (83.3)	7 (70.0)
Migraine with aura (*n*, [%])	7 (25.0)	3 (25.0)	1 (16.7)	3 (30.0)
Medication overuse headache (*n*, [%])	14 (50.0)	6 (50.0)	3 (50.0)	4 (50.0)
Onset, years	23.5 ± 8.1	22.4 ± 7.5	27.0 ± 5.5	22.6 ± 10.0
Disease history, years	20.0 ± 7.9	22.3 ± 6.0	18.8 ± 6.3	18.0 ± 10.5
Disease duration from the first visit, years	5.58 ± 4.08	5.92 ± 4.08	6.42 ± 5.08	4.58 ± 3.58

Data are presented as mean ± SD or *n* (%), as appropriate. Percentages are calculated within each column unless otherwise indicated.

EM, episodic migraine; HFEM, high-frequency episodic migraine; CM, chronic migraine; GAL, galcanezumab; FRE(M), fremanezumab monthly; FRE(Q), fremanezumab quarterly.

*HFEM is presented as a subset within EM (i.e., EM includes HFEM); CM is shown separately.

*N* at baseline: 50 (GAL 24; FRE(M) 10; FRE(Q) 16); *N* among 3-year continuers: 28 (GAL 12; FRE(M) 6; FRE(Q) 10).

**Table 2:** Baseline characteristics by LFEM (0–7 days), HFEM (8–14 days), and CM (≥15 days).

Patient group	LFEM (0–7 days)	HFEM (8–14 days)	CM (≥15 days)
Number of patients	13	20	17
Age, years	43.8 ± 10.0	42.5 ± 11.5	41.6 ± 13.7
Sex, female (%)	10 (76.9)	18 (90.0)	16 (94.1)
Migraine without aura (*n*, [%])	9 (69.2)	16 (80.0)	12 (70.6)
Migraine with aura (*n*, [%])	4 (30.8)	4 (20.0)	5 (29.4)
Medication overuse headache (*n*, [%])	4 (30.8)	10 (50.0)	11 (64.7)
Onset, years	22.5 ± 10.2	22.3 ± 8.1	22.4 ± 6.0
Disease history, years	21.3 ± 8.4	20.2 ± 10.2	19.2 ± 10.8
Disease duration from the first visit, years	4.42 ± 3.33	5.42 ± 3.92	4.17 ± 4.75
Outcome			
Continuous anti-CGRP use (3 years, *n* [%])	7 (53.8)	13 (65.0)	8 (47.0)
Self-discontinuation (3 years, *n* [%])	3 (23.1)	2 (10.0)	1 (5.9)
Planned discontinuation (treatment completion) (3 years, *n* [%])	1 (7.7)	4 (20.0)	7 (41.2)
Anti-CGRP switch (3 years, *n* (%)), e.g., FRE(M) → GAL, GAL→FRE(Q)	1 (7.7), GAL→FRE(Q)	0 (0.0)	0 (0.0)
Referral (3 years, *n* [%])	1 (7.7)	1 (5.0)	1 (5.9)

Continuous anti-CGRP use (3 years, *n*)	7	13	8
Age, years	42.0 ± 9.3	43.6 ± 8.9	44.5 ± 11.2
Sex, female (%)	5 (71.4)	11 (84.6)	8 (100.0)
Migraine without aura (*n*, [%])	4 (57.1)	12 (92.3)	7 (87.5)
Migraine with aura (*n*, (%))	3 (42.9)	1 (7.7)	5 (62.5)
Medication overuse headache (*n*, [%])	2 (28.6)	6 (46.2)	3 (37.5)
Onset, years	22.6 ± 10.9	23.6 ± 8.3	24.0 ± 5.8
Disease history, years	19.4 ± 7.7	20.0 ± 8.1	20.5 ± 9.0
Disease duration from the first visit, years	5.08 ± 2.58	5.33 ± 3.75	6.25 ± 5.67

### 3.2 Treatment persistence

**Figure 1:**
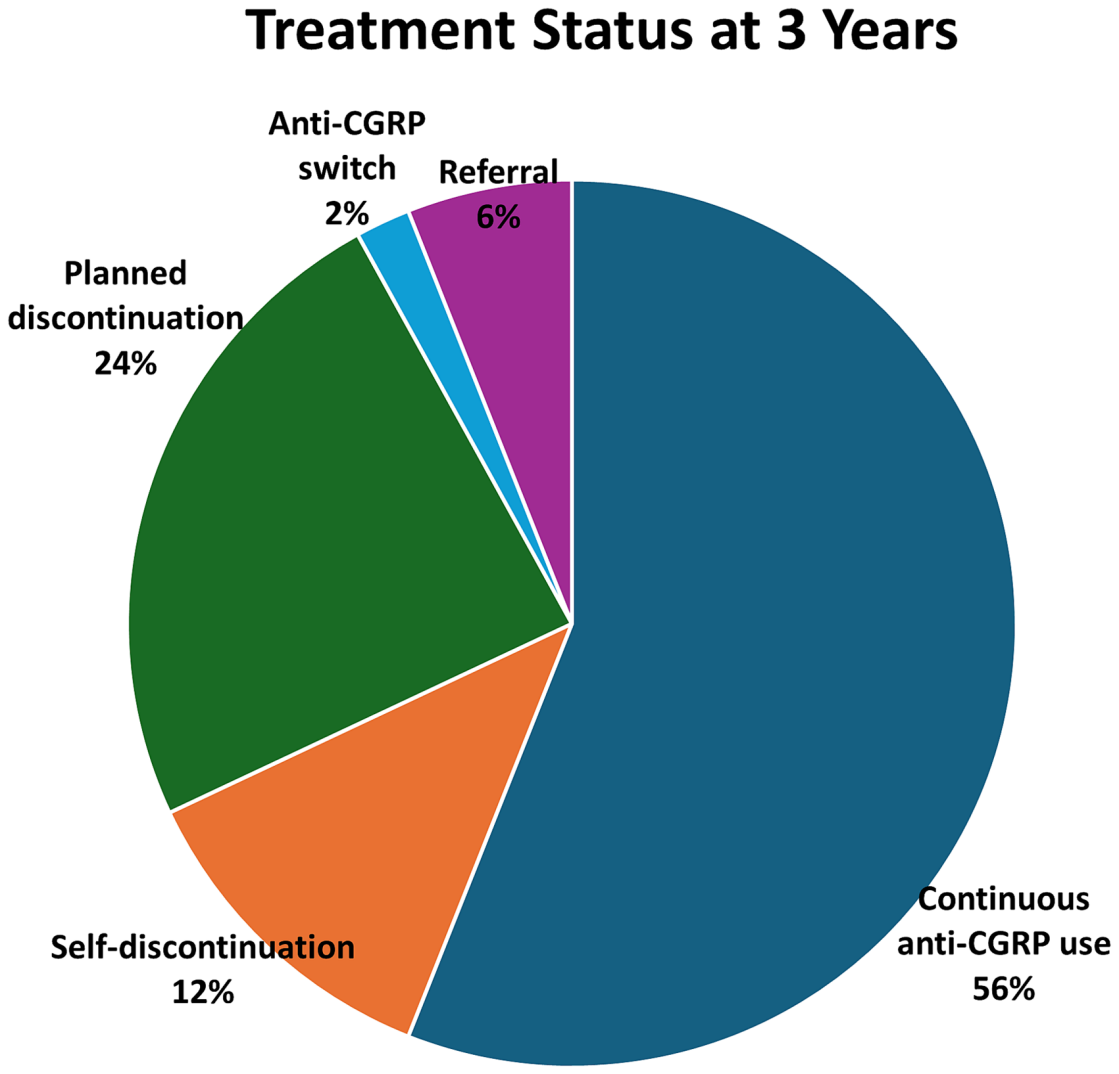
Treatment continuation and reasons for discontinuation over 3 years. Of the 50 patients who initiated anti-CGRP monoclonal antibody therapy, 28 of 50 (56.0%) remained on treatment at 36 months. Reasons for discontinuation among the remainder were treatment completion after goal attainment (12/50, 24.0%), self-discontinuation (6/50, 12.0%), referral (3/50, 6.0%), and switching to another CGRP agent (1/50, 2.0%). No discontinuations were observed due to adverse events (0/50). Treatment completion was defined *a priori* as planned discontinuation following goal attainment (a ≥ 50% reduction in MMDs sustained across ≥ 2 consecutive assessments, based on shared decision-making). Numbers at risk at each time point are provided in Supplementary Table S2. CGRP, calcitonin gene-related peptide. Proportions at 36 months are calculated using the baseline cohort (*n* = 50) as the denominator.

At 36 months, 28 of the 50 patients (56.0%) remained on therapy. Among the 22 discontinuations, treatment completion according to the predefined criteria occurred in 12 patients (24.0%), self-discontinuation in 6 (12.0%), referral in 3 (6.0%), and switching in 1 (2.0%); no discontinuations were attributed to adverse events. A single regimen switch was recorded at the 12-month visit (galcanezumab→fremanezumab quarterly), with no additional switches observed. Numbers at risk and evaluable denominators at each time point are provided in the figure and table footnotes and in Supplementary Table S2 and Figure 1.

All cases classified as treatment completion met the predefined criterion of a ≥ 50% reduction in MMDs sustained across ≥2 consecutive scheduled assessments, accompanied by patient–clinician shared decision-making to pause anti-CGRP therapy.

Retention at scheduled visits was 48 of 50 at 1 month, 48 of 50 at 3 months, 42 of 50 at 6 months, 36 of 50 at 1 year, and 28 of 50 at 3 years (cumulative discontinuations: 2, 2, 8, 14, and 22, respectively). Interval discontinuations comprised treatment completion (0, 0, 1, 3, 8), self-discontinuation (2, 0, 2, 2, 0), referral (0, 0, 3, 0, 0), and switching to another CGRP agent (0, 0, 0, 1, 0). No discontinuations were observed due to adverse events (Supplementary Table S2).

Planned discontinuation (“treatment completion”) occurred in 5 of 33 (15.2%) patients with EM (LFEM+HFEM) and 11 of 37 (29.7%) patients with HFEM+CM; the difference was not statistically significant (Fisher’s exact *p* = 0.17).

Non-serious adverse events were limited to injection-site reactions (pain, *n* = 5; erythema, *n* = 8), with an overlap observed in 3 patients; no AE-related discontinuations occurred (Supplementary Table S4). Visit-level denominators for the baseline cohort are provided in Supplementary Table S2.

### 3.3 Clinical outcomes

**Figure 2:**
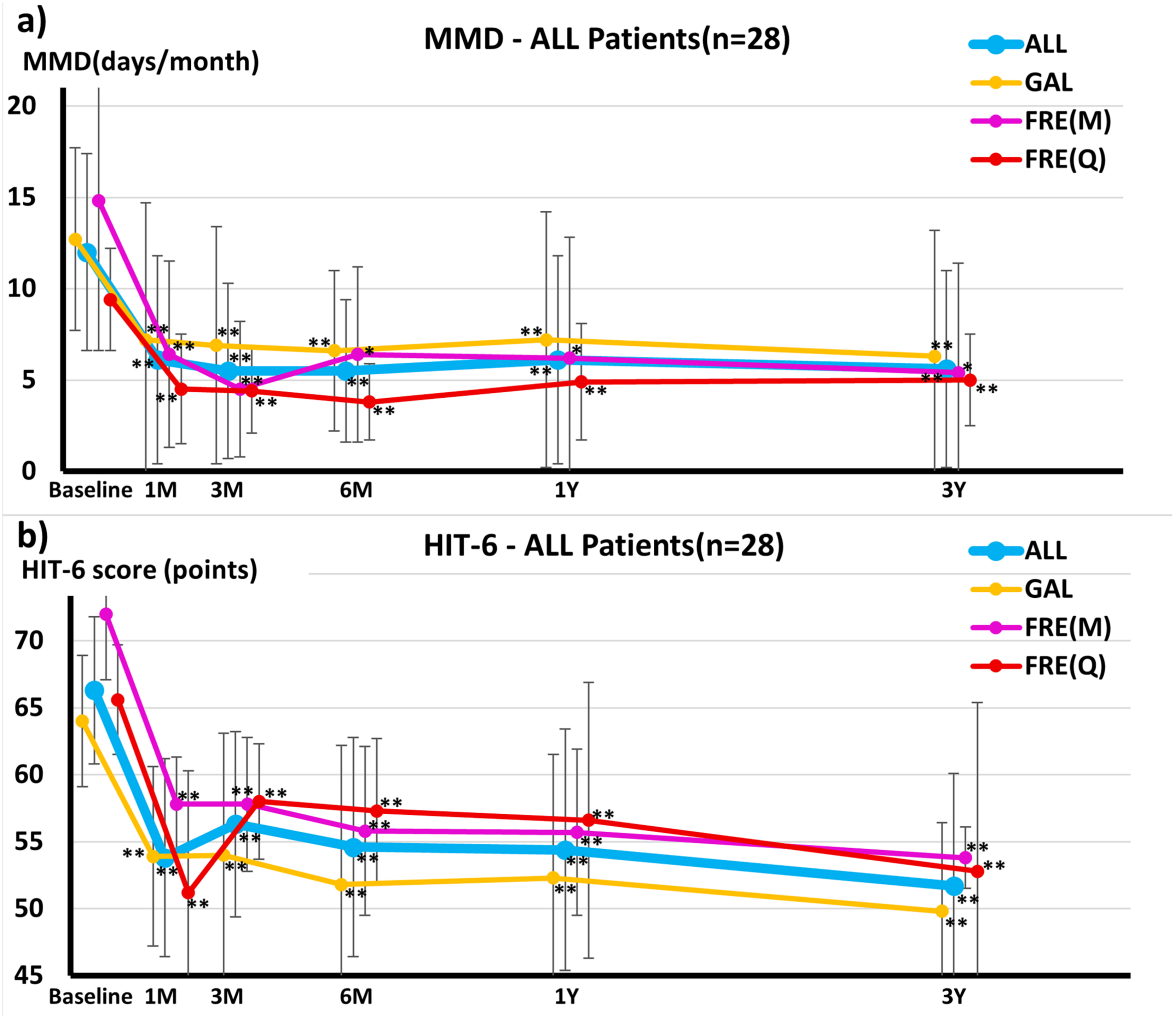
Changes in **(a)** monthly migraine days (MMDs) and **(b)** Headache Impact Test (HIT-6) scores from baseline to 1, 3, 6, 12, and 36 months. Lines indicate ALL (overall cohort), GAL (galcanezumab), FRE(M) (fremanezumab monthly), and FRE(Q) (fremanezumab quarterly). Symbols/line types are differentiated for monochrome print: ALL (solid circle, solid line), GAL (open circle, dashed line), FRE(M) (solid triangle, dotted line), and FRE(Q) (open triangle, dash-dot line). Error bars represent SD. *p < 0.05; **p < 0.01 vs. baseline (paired *t*-test). MMD, monthly migraine days; HIT-6, Headache Impact Test-6; GAL, galcanezumab; FRE(M), fremanezumab monthly; FRE(Q), fremanezumab quarterly; SD, standard deviation. Note: The sample size (n) at each time point is provided; denominators vary by time point due to data availability. Detailed trajectories for all other outcome panels are provided in Supplementary Figure S1.

Across the cohort continuing to 3 years (*n* = 28), all key measures improved significantly from baseline to month 36 (Figure 2 and Table 3): MMDs decreased from 12.0 ± 5.4 to 5.6 ± 5.4 (*p* < 0.01); MIDAS scores improved from 33.1 ± 34.4 to 9.1 ± 18.1 (*p* < 0.01); HIT-6 scores decreased from 66.3 ± 5.5 to 51.7 ± 8.4 (*p* < 0.01); and VAS scores decreased from 76.4 ± 17.4 to 29.8 ± 22.6 (*p* < 0.01). Detailed time series for other outcomes are shown in Supplementary Figure S1.

**Table 3:** Clinical outcomes by treatment (galcanezumab/fremanezumab monthly/fremanezumab quarterly).

Outcome	Treatment group	Baseline	1 month	3 months	6 months	1 year	3 years
All patients (*n* = 28)							
MMDs	All products	12.0 ± 5.4	6.1 ± 5.7^**^	5.5 ± 4.8^**^	5.5 ± 3.9^**^	6.1 ± 5.7^**^	5.6 ± 5.4^**^
	Galcanezumab	12.7 ± 5.0	7.2 ± 7.5^**^	6.9 ± 6.5^**^	6.6 ± 4.4^**^	7.2 ± 7.0^**^	6.3 ± 6.9^**^
	Fremanezumab Monthly	14.8 ± 8.2	6.4 ± 5.1^**^	4.5 ± 3.7^**^	6.4 ± 4.8^*^	6.2 ± 6.6^*^	5.4 ± 6.0^*^
	Fremanezumab Quarterly	9.4 ± 2.8	4.5 ± 3.0^**^	4.4 ± 2.3^**^	3.8 ± 2.1^**^	4.9 ± 3.2^**^	5.0 ± 2.5^**^
MIDAS	All products	33.1 ± 34.4	23.7 ± 39.3 ^N. S.^	11.0 ± 17.2^**^	13.0 ± 14.7^**^	11.7 ± 16.6^**^	9.1 ± 18.1^**^
	Galcanezumab	17.6 ± 18.6	16.3 ± 14.4 ^N. S.^	8.5 ± 10.1^*^	10.9 ± 13.0^*^	6.1 ± 8.5^*^	3.4 ± 4.8^*^
	Fremanezumab Monthly	73.8 ± 49.9	57.3 ± 76.7 ^N. S.^	24.3 ± 31.6^**^	21.0 ± 19.6^**^	18.2 ± 16.1^*^	8.7 ± 6.2^*^
	Fremanezumab Quarterly	27.4 ± 16.5	12.5 ± 12.2^*^	6.1 ± 7.0^**^	10.6 ± 13.3^**^	14.9 ± 23.4^*^	17.1 ± 29.8^*^
HIT-6	All products	66.3 ± 5.5	53.8 ± 7.4^**^	56.3 ± 6.9^**^	54.6 ± 8.2^**^	54.4 ± 9.0^**^	51.7 ± 8.4^**^
	Galcanezumab	64.0 ± 4.9	53.9 ± 6.7^**^	54.0 ± 9.1^**^	51.8 ± 10.4^**^	52.3 ± 9.2^**^	49.8 ± 6.6^**^
	Fremanezumab Monthly	72.0 ± 4.9	57.8 ± 3.5^**^	57.8 ± 5.0^**^	55.8 ± 6.3^**^	55.7 ± 6.2^**^	53.8 ± 2.3^**^
	Fremanezumab Quarterly	65.6 ± 4.1	51.2 ± 9.1^**^	58.0 ± 4.3^**^	57.3 ± 5.4^**^	56.6 ± 10.3^**^	52.8 ± 12.6^**^
VAS	All products	76.4 ± 17.4	40.3 ± 27.1^**^	36.2 ± 22.6^**^	35.0 ± 22.9^**^	36.9 ± 25.0^**^	29.8 ± 22.6^**^
	Galcanezumab	69.9 ± 20.9	38.6 ± 25.3^**^	36.0 ± 23.5^**^	30.7 ± 25.6^**^	28.9 ± 24.4^**^	20.0 ± 15.6^**^
	Fremanezumab Monthly	82.3 ± 17.1	49.3 ± 35.7^**^	33.5 ± 22.0^**^	37.3 ± 26.9^**^	46.5 ± 27.6^**^	36.3 ± 25.0^**^
	Fremanezumab Quarterly	80.6 ± 10.6	37.0 ± 25.3^**^	38.0 ± 24.1^**^	38.7 ± 18.3^**^	41.8 ± 23.0^**^	38.4 ± 25.9^**^
Patients with EM (*n* = 20)							
MMDs	All products	9.6 ± 2.8	5.0 ± 3.5^**^	5.1 ± 3.1^**^	5.9 ± 3.5^**^	5.3 ± 3.3^**^	4.5 ± 2.7^**^
	Galcanezumab	9.8 ± 3.0	5.1 ± 4.7^**^	6.2 ± 4.1^*^	7.9 ± 4.1^N. S.^	5.6 ± 4.2^**^	3.8 ± 2.9^**^
	Fremanezumab Monthly	11.3 ± 4.1	4.4 ± 3.5^*^	3.4 ± 2.3^**^	6.0 ± 3.6^**^	4.5 ± 1.4^*^	3.2 ± 3.8^*^
	Fremanezumab Quarterly	8.8 ± 2.1	5.0 ± 2.7^**^	4.8 ± 2.1^**^	4.1 ± 2.0^**^	5.3 ± 3.1^**^	5.6 ± 1.7^**^
MIDAS	All products	25.5 ± 19.2	13.2 ± 12.1^**^	7.9 ± 9.2^**^	11.2 ± 13.0^**^	11.4 ± 18.0^**^	11.3 ± 21.1^**^
	Galcanezumab	19.1 ± 20.5	14.9 ± 13.6 ^N. S.^	10.1 ± 12.1 ^N. S.^	12.4 ± 14.4^*^	5.8 ± 10.1 ^N. S.^	4.6 ± 5.4^*^
	Fremanezumab Monthly	31.7 ± 25.5	9.3 ± 7.2 ^N. S.^	6.0 ± 7.0 ^N. S.^	6.7 ± 9.0 ^N. S.^	14.0 ± 14.4^N. S.^	7.7 ± 9.0 ^N. S.^
	Fremanezumab Quarterly	29.1 ± 16.5	12.9 ± 12.8^*^	6.6 ± 7.2^*^	11.6 ± 13.7^**^	16.1 ± 24.7^*^	19.3 ± 31.1^*^
HIT-6	All products	66.3 ± 4.7	53.2 ± 8.0^**^	56.9 ± 5.5^**^	55.7 ± 7.3^**^	52.7 ± 9.4^**^	52.7 ± 8.3^**^
	Galcanezumab	65.6 ± 3.2	55.3 ± 7.3^**^	56.9 ± 7.1^**^	55.3 ± 9.3^**^	50.8 ± 10.2^**^	50.8 ± 5.7^**^
	Fremanezumab Monthly	71.7 ± 7.8	57.0 ± 3.6^*^	54.3 ± 4.5^*^	53.3 ± 8.1^**^	51.3 ± 6.1^**^	52.3 ± 2.1^*^
	Fremanezumab Quarterly	65.1 ± 4.1	50.1 ± 9.0^**^	57.8 ± 4.5^**^	56.9 ± 5.6^**^	55.1 ± 10.1^**^	54.9 ± 11.6^**^
VAS	All products	77.3 ± 16.3	39.0 ± 28.3^**^	37.8 ± 21.9^**^	35.7 ± 21.4^**^	33.3 ± 23.2^**^	29.2 ± 22.1^**^
	Galcanezumab	73.3 ± 21.7	36.5 ± 26.3^*^	39.6 ± 20.1^**^	35.8 ± 24.9^**^	27.3 ± 24.7^**^	18.0 ± 10.8^**^
	Fremanezumab Monthly	80.7 ± 16.8	50.0 ± 45.8 ^N. S.^	27.7 ± 21.1^**^	30.0 ± 26.5^**^	35.3 ± 21.6^**^	30.0 ± 21.1^*^
	Fremanezumab Quarterly	79.8 ± 11.0	37.5 ± 26.8^*^	39.6 ± 25.1^**^	37.4 ± 18.9^**^	39.4 ± 23.9^**^	40.1 ± 27.2^**^
Patients with HFEM or CM (*n* = 21)							
MMDs	All products	13.8 ± 5.2	7.2 ± 6.0^**^	6.0 ± 5.2^**^	6.0 ± 4.1^**^	6.9 ± 6.2^**^	6.3 ± 5.7^**^
	Galcanezumab	14.0 ± 4.4	8.3 ± 7.8^*^	7.1 ± 7.0^**^	6.6 ± 4.5^**^	8.2 ± 7.3^*^	7.5 ± 6.9^**^
	Fremanezumab Monthly	16.5 ± 7.9	7.5 ± 4.8^**^	5.3 ± 3.6^*^	7.3 ± 4.8^*^	6.7 ± 7.2^*^	6.2 ± 6.3^*^
	Fremanezumab Quarterly	11.1 ± 2.3	5.3 ± 3.1^*^	4.8 ± 2.5^*^	3.9 ± 2.0^**^	4.8 ± 3.1^**^	4.5 ± 2.7^**^
MIDAS	All products	36.9 ± 38.2	28.9 ± 44.0 ^N. S.^	14.0 ± 19.0^**^	13.9 ± 14.6^**^	11.3 ± 12.9^**^	7.2 ± 9.8^**^
	Galcanezumab	19.8 ± 19.8	16.9 ± 14.9 ^N. S.^	10.1 ± 10.4 ^N. S.^	12.8 ± 13.5 ^N. S.^	7.3 ± 8.8 ^N. S.^	3.7 ± 5.2^*^
	Fremanezumab Monthly	82.6 ± 50.4	68.6 ± 80.0 ^N. S.^	29.0 ± 33.0^*^	24.8 ± 19.3^**^	19.8 ± 17.4^*^	10.0 ± 5.9^*^
	Fremanezumab Quarterly	27.3 ± 17.6	15.8 ± 12.2 ^N. S.^	8.0 ± 8.5^*^	6.7 ± 6.6^*^	10.8 ± 13.4^*^	10.7 ± 16.1^*^
HIT-6	All products	66.7 ± 5.6	55.1 ± 6.2^**^	56.3 ± 7.8^**^	55.9 ± 8.6^**^	55.7 ± 8.7^**^	51.0 ± 7.8^**^
	Galcanezumab	63.7 ± 5.4	54.0 ± 7.3^**^	54.2 ± 10.0^**^	53.4 ± 10.8^**^	54.5 ± 8.4^*^	49.5 ± 6.5^**^
	Fremanezumab Monthly	71.6 ± 5.4	58.6 ± 3.4^**^	57.6 ± 5.5^**^	56.6 ± 6.7^**^	56.8 ± 6.1^**^	54.0 ± 2.5^**^
	Fremanezumab Quarterly	67.5 ± 2.8	54.0 ± 5.9^**^	58.8 ± 4.8^**^	59.5 ± 5.2^**^	56.8 ± 11.9^*^	50.8 ± 12.2^**^
VAS	All products	74.4 ± 18.5	43.7 ± 28.2^**^	38.7 ± 21.2^**^	37.0 ± 23.5^**^	38.2 ± 25.8^**^	29.8 ± 20.6^**^
	Galcanezumab	66.4 ± 20.4	41.8 ± 26.6^*^	37.2 ± 25.3^**^	32.3 ± 27.9^**^	31.2 ± 26.3^**^	20.5 ± 16.8^**^
	Fremanezumab Monthly	84.3 ± 18.3	57.2 ± 33.6^*^	38.6 ± 20.2^**^	42.8 ± 26.1^**^	50.6 ± 28.8^**^	42.0 ± 23.3^**^
	Fremanezumab Quarterly	79.6 ± 9.5	35.8 ± 26.9^*^	41.3 ± 17.6^**^	40.0 ± 13.4^**^	39.8 ± 21.3^**^	35.2 ± 20.1^**^

**p < 0.01 vs. baseline; *p < 0.05 vs. baseline; N. S., not significant vs. baseline.

MMDs, monthly migraine days; MIDAS, Migraine Disability Assessment Scale; HIT-6, Headache Impact Test; VAS, Visual Analogue Scale; EM, episodic migraine; HFEM, high-frequency episodic migraine; CM, chronic migraine.

Table presents the cohort continuing to 3 years (*n* = 28); denominators are constant across visits in this table.

### 3.4 RRs

**Figure 3:**
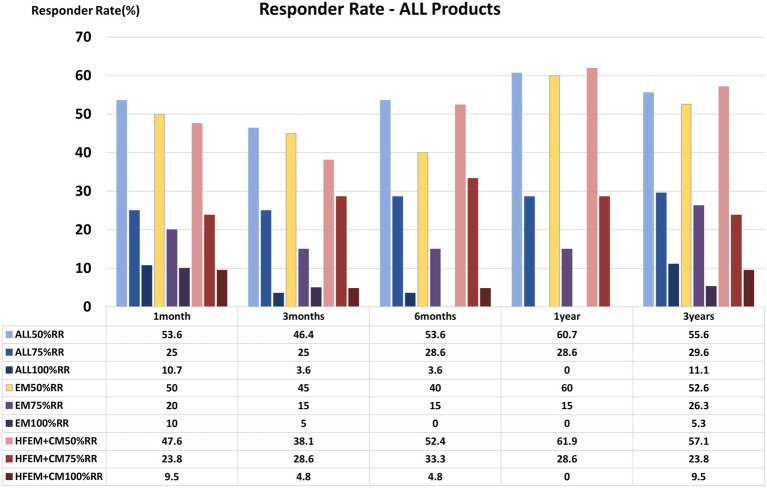
Responder rates (RRs) over time for the overall cohort and subgroups. Proportions achieving ≥50%, ≥75, and 100% reduction in monthly migraine days are shown at 1, 3, 6, 12, and 36 months. Comparisons between EM (including HFEM) and CM at each time point used the chi-squared test (*p < 0.05). Abbreviations: EM, episodic migraine; HFEM, high-frequency episodic migraine (subset within EM); CM, chronic migraine. Note: The sample size (n) at each time point is provided; denominators vary by time point due to data availability (e.g., the 36-month RR calculated among patients with evaluable data at 36 months). Additional regimen-specific responder panels are provided in Supplementary Figure S2.

Overall RRs indicated durable benefit (Figure 3 and Supplementary material): ≥50% RRs, 53.6% at 1 month and 55.6% at 3 years (stable over time); ≥75% RRs increased modestly over time (25.0% → 29.6%); and 100% RRs, remained low overall (11.1% at 3 years), consistent with the multifactorial nature of migraine. Regimen-specific responder panels are provided in Supplementary Figure S2.

### 3.5 Subgroups and regimens

No significant differences were observed between EM and HFEM+CM for primary outcomes; EM tended to exhibit earlier improvement, aligning with prior reports. Across treatment regimen, all groups showed significant within-group improvements. At 3 years, a greater VAS reduction was observed in the galcanezumab group than in other groups (*p* = 0.04), while the fremanezumab monthly group achieved the highest ≥75% RRs (50%). Detailed drug- and dosing-specific trajectories are provided in the Supplementary material to streamline the main text.

### 3.6 Sensitivity analyses

Sensitivity analyses using a GLMM with a Gaussian distribution and identity link, incorporating a patient-level random intercept, together with multiple imputation (*m* = 50), yielded time-point changes and corresponding 95% CIs that were directionally consistent with the primary analyses. These findings support the robustness of the results to missing outcomes and potential continuation bias (Supplementary Table S3). Full model estimates are provided in Supplementary Table S3.

## 4 Discussion

To the best of our knowledge, this is among the first few studies to provide 3-year real-world data on anti-CGRP monoclonal antibodies in Japan and one of the longest follow-ups reported globally. Such long-term evidence is rare and critical for guiding treatment continuation, discontinuation strategies, and regimen selection in routine clinical practice.

Our findings demonstrate that anti-CGRP monoclonal antibodies provide sustained preventive benefit over 3 years, with significant and durable reductions in MMDs and clinically significant improvements across disability (MIDAS), daily impact (HIT-6), and pain intensity (VAS). The 56% 3-year continuation rate and the predominance of treatment completion—rather than adverse events—as the principal discontinuation reason highlight favorable tolerability, perceived effectiveness, and attainment of patient-specific goals in routine practice. These findings are directionally consistent with evidence from randomized trials on galcanezumab and fremanezumab in EM and CM, which reported robust reductions in attack frequency and improvements in PROs (1–6).

From a care delivery perspective, our results extend the evidence base beyond the 12–24-month horizon commonly reported in early RWE, adding longitudinal data on persistence and discontinuation reasons (7–9). The stability of the ≥50% RR and incremental gains in the ≥75% RR suggest that some patients accrue greater benefit with continued therapy, potentially reflecting cumulative reductions in central sensitisation and improved self-management behaviors over time. Conversely, the low overall 100% RR underscores that complete remission remains uncommon even with sustained CGRP blockade. This is consistent with the multifactorial nature of migraine and the observations from trials, which posit that only a significant minority of people achieve very high response thresholds (1–6). Subgroup patterns were clinically intuitive: patients with EM tended to respond earlier than those with HFEM+CM, a trend consistent with evidence from randomized trials (EVOLVE-2, REGAIN, HALO-EM/CM), which collectively suggest that baseline burden and central sensitisation may influence trajectory and time to response (2, 4–6). They also align with findings from Japanese real-world cohorts, including our prior studies focusing on assessment tools and outcomes among anti-CGRP-eligible patients (7–9, 14). Regimen-level signals—greater VAS reduction with galcanezumab and higher ≥75% RR with monthly fremanezumab at 3 years—should be interpreted cautiously given the sample size and observational design of our study. Nonetheless, they echo real-world dosing reports in Japan showing comparable effectiveness across fremanezumab schedules and provide hypotheses for individualized selection that merit prospective evaluation (7, 9, 15). Our primary EM and HFEM+CM grouping was prespecified to reflect preventive care decision-making, with HFEM/CM representing a high-burden, preventive-eligible cluster. This approach is supported by recent literature identifying HFEM as a distinct, high-burden subset within the episodic migraine spectrum. Furthermore, CGRP-targeting therapies are endorsed as first-line preventive options across the EM-to-CM continuum (13), and real-world evidence on treatment persistence supports their practical implementation (16–19).

Optimal treatment duration and long-term safety remain practical challenges in CGRP-targeting prevention. Recent reviews highlight that, while CGRP-pathway therapies show favorable efficacy and tolerability, important questions remain regarding individualized treatment length, stopping strategies, and monitoring during prolonged exposure (20). In addition, evidence syntheses focusing on ≥12 months of exposure suggest that discontinuation due to adverse events is generally low, with largely stable and predominantly non-serious adverse-event profiles over extended follow-up. These data provide context for our real-world observation of no adverse-event-related discontinuations while underscoring the importance of systematic safety documentation and ongoing surveillance in routine long-term care (21).

Importantly, persistence and completion patterns observed here complement the broader literature on adherence challenges with older oral preventives, where tolerability, adverse effects, and limited efficacy often curtailed long-term use (22–26). In contrast, anti-CGRP antibodies support longer-term continuation and goal-directed discontinuation in routine practice, as also suggested by multicenter real-world studies emphasizing early initiation and sustained benefit early treatment with anti-CGRP monoclonal antibodies enhances response in the real world (EUREkA) (27). Together, these data inform counseling about treatment continuation, dose scheduling, and planned cessation—areas where standardized criteria remain limited in everyday care (10).

### 4.1 Clinical implications and future directions

In addition to demonstrating sustained efficacy and tolerability, these data provide practical guidance for clinicians, helping them decide on continuation vs. planned discontinuation and regimen selection in everyday practice. Such insights are essential for optimizing long-term migraine management in real-world settings.

Future studies should incorporate multicenter prospective cohorts, explicit and harmonized discontinuation criteria (e.g., sustained MMD thresholds and PRO stability), predictors of sustained response (clinical and biomarker-based), and comprehensive assessments of quality of life, functioning, and cost-effectiveness—directions that are consistent with the International Headache Society (IHS) recommendations for RWE in migraine (10).

### 4.2 Limitations

First, the overall sample size was small (*n* = 50) in this single-center cohort, limiting precision and statistical power for detecting between-group differences. This single-center, retrospective RWE design entails risks of selection and information biases. The outcomes relied on patient diaries/smartphone applications, which may introduce reporting bias despite verification at clinic visits. Dose-defined subgroups were modest, and we did not standardize the evaluation of concomitant medications or cost-related factors, leaving room for unmeasured confounding. Adherence metrics (e.g., on-time dosing) were not systematically recorded beyond routine visit verification and therefore could not be quantified; accordingly, we report visit-level persistence with transparent denominators at each time point. In addition, treatment discontinuation decisions were based on an operational real-world criterion rather than a fixed protocol, which may limit standardization. External validity may be constrained because all patients were managed in a Japanese tertiary care setting under national prescribing and reimbursement rules; differences in healthcare systems, patient mix, and access may alter long-term outcomes elsewhere. Nevertheless, prespecified sensitivity analyses—including a GLMM (Gaussian/identity with a patient-level random intercept) and multiple imputation (*m* = 50)—supported the robustness of the primary findings. These considerations mirror the IHS guidelines for RWE in migraine (10) and reinforce priorities for future research: multi-center prospective cohorts with harmonized discontinuation criteria (e.g., sustained MMD/PRO thresholds), predictors of sustained response (clinical/biomarker-based), and full assessments of quality of life, functioning, and cost-effectiveness (7–9, 14, 15, 27).

### 4.3 Conclusion

Anti-CGRP monoclonal antibodies provided sustained preventive efficacy and favorable tolerability over 3 years, with overall improvements of approximately 40% in MMDs and 18% in HIT-6 scores. Galcanezumab achieved a 42% reduction in MMDs and 22% in the HIT-6, while fremanezumab showed similar benefits, with the monthly regimen yielding the greatest improvement (MMDs −67%, HIT-6 − 26%). Pain intensity reduction was most pronounced with galcanezumab at 3 years (VAS, *p* = 0.04). Although early treatment gains (−47 to −60% in MMD at 1–6 months) attenuated slightly over time, clinically significant benefits were maintained across all regimens. These findings support anti-CGRP monoclonal antibodies as durable preventive options and highlight the need for individualized dosing strategies and clear discontinuation criteria. Together, these data help clinicians decide on treatment continuation vs. discontinuation and regimen selection in everyday practice.

## Data Availability

De-identified data supporting the findings of this study, together with the analysis code for sensitivity analyses, are available from the corresponding author (for academic, non-commercial use) upon reasonable request and subject to a data use agreement and applicable institutional and ethical regulations. Data are not publicly available due to privacy and ethical restrictions. Aggregate tables and detailed model outputs are provided in the Supplementary material.
