# Supplementary material for: Three-year real-world effectiveness, treatment persistence, and planned discontinuation of anti-calcitonin gene-related peptide monoclonal antibodies for migraine prevention: a single-center cohort from Japan
Source: Front Neurol. 2026 May 13;17:1827022. doi: 10.3389/fneur.2026.1827022 (PMC13212110; doi:10.3389/fneur.2026.1827022)
Supplement: Supplementary file 1 [file Supplementary_file_1.docx]

Supplementary Material

# Supplementary Figures and Tables

## Supplementary Figures

**S1a)**

**S1b)**

**S1c)**

**S1d)**

**S1e)**

**S1f)**

**S1g)**

**S1h)**

**S1i)**

**S1j)**

**Supplementary Figure S1. Detailed longitudinal trajectories for primary and secondary outcomes.**

Panels: (S1a) MMDs in EM; (S1b) MMDs in HFEM+CM; (S1c–e) MIDAS; (S1f–g) HIT‑6; (S1h–j) VAS.

Lines: ALL (solid circle, solid line), GAL (open circle, dashed line), FRE(M) (solid triangle, dotted line), FRE(Q) (open triangle, dash‑dot line). Error bars represent SD.

**p<0.01; *p<0.05 vs. baseline (paired t‑test).

Abbreviations: MMD, monthly migraine days; MIDAS, Migraine Disability Assessment Scale; HIT-6, Headache Impact Test-6; VAS, visual analog scale; GAL, galcanezumab; FRE(M), fremanezumab monthly; FRE(Q), fremanezumab quarterly; SD, standard deviation; EM, episodic migraine; HFEM, high-frequency episodic migraine; CM, chronic migraine.

Note: Denominators vary by time point and subgroup; EM includes HFEM.

**S2a)**

**S2b)**

**S2c)**

**Supplementary Figure S2. Responder rates (≥50%, ≥75%, 100%) by regimen and subgroup at 1, 3, 6, 12, and 36 months.**

Panels: (S2a) GAL; (S2b) FRE(M); (S2c) FRE(Q). Subpanels compare EM (including HFEM) and CM at each time point (chi‑square test).

Abbreviations: RR, responder rate; MMD, monthly migraine days; GAL, galcanezumab; FRE(M), fremanezumab monthly; FRE(Q), fremanezumab quarterly; EM, episodic migraine; HFEM, high-frequency episodic migraine (subset within EM); CM, chronic migraine.

Note: Denominators vary by time point and regimen; percentages are shown with the available n at each time point.

**1.2 Supplementary Tables**

**Supplementary Table S1. STROBE reporting checklist for observational cohort studies used to guide reporting in this manuscript.** Note: Adapted from the STROBE reporting checklist. References: (1) von Elm E, Altman DG, Egger M, et al. Ann Intern Med. 2007;147(8):573–577. (2) The STROBE reporting checklist, EQUATOR Network.

|  | Item Description | Location (or reason for not reporting) |
| --- | --- | --- |
| **Title and abstract** |  |  |
| 1a. Indicate the study’s design | Indicate the study’s design with a commonly used term in the title or the abstract. | Title; Abstract (first sentence) |
| 1b. Abstract | Provide in the abstract an informative and balanced summary of what was done and what was found. | Abstract, entire section |
| **Introduction** |  |  |
| 2. Background / rationale | Explain the scientific background and rationale for the investigation being reported. | Introduction, paragraph 1 |
| 3. Objectives | State specific objectives, including any prespecified hypotheses. | Introduction, last paragraph |
| **Methods** |  |  |
| 4. Study design | Present key elements of study design early in the paper. | Methods, paragraph 1 |
| 5. Setting | Describe the setting, locations, and relevant dates, including periods of recruitment, exposure, follow-up, and data collection. | Methods, paragraph 1 |
| 6a. Eligibility criteria | **Cohort study:** Give the eligibility criteria, and the sources and methods of selection of participants. Describe methods of follow-up. **Case-control study:** Give the eligibility criteria, and the sources and methods of case ascertainment and control selection. Give the rationale for the choice of cases and controls. **Cross-sectional study:** Give the eligibility criteria, and the sources and methods of selection of participants. | Methods, paragraph 2 |
| 6b. Matching criteria | **Cohort study:** For matched studies, give matching criteria and number of exposed and unexposed. **Case-control study:** For matched studies, give matching criteria and the number of controls per case. |  |
| 7. Variables | Clearly define all outcomes, exposures, predictors, potential confounders, and effect modifiers. Give diagnostic criteria, if applicable. | Methods, paragraph 3 |
| 8. Data sources / measurement | For each variable of interest give sources of data and details of methods of assessment (measurement). Describe comparability of assessment methods if there is more than one group. | Methods, paragraph 3 |
| 9. Bias | Describe any efforts to address potential sources of bias. | Methods — RWE design and reporting (data provenance/verification); Statistical analysis and handling of bias (GLMM [Gaussian/identity; patient-level random intercept], multiple imputation [m=50], prespecified sensitivity analyses). |
| 10. Study size | Explain how the study size was arrived at. | Methods, paragraph 2 |
| 11. Quantitative variables | Explain how quantitative variables were handled in the analyses. If applicable, describe which groupings were chosen, and why. | Methods, paragraph 4 |
| 12a. Statistical methods | Described GLMM (Gaussian/identity; random intercept for patient; Time categorical) and multiple imputation (m=50) for sensitivity analyses. | Methods, paragraph 4 |
| 12b. Statistical methods – subgroups and interactions | Describe any methods used to examine subgroups and interactions. | Methods, paragraph 4 |
| 12c. Statistical methods – missing data | Multiple imputation (m=50) under MAR; model‑based estimates from GLMM. | Methods, paragraph 3 |
| 12di. Statistical methods – loss to follow-up | **Cohort study:** If applicable, describe how loss to follow-up was addressed. | Methods — Statistical analysis: no loss to follow-up; participants discontinuing treatment had no further scheduled assessments per protocol. |
| 12dii. Statistical methods – matching cases and controls | **Case-control study:** If applicable, explain how matching of cases and controls was addressed. |  |
| 12diii. Statistical methods – sampling strategy | **Cross-sectional study:** If applicable, describe analytical methods taking account of sampling strategy. |  |
| 12e. Statistical methods – sensitivity analyses | Results summarized in Supplementary Table S3. | Results, paragraph 3 |
| **Results** |  |  |
| 13a. Participant numbers | Report the numbers of individuals at each stage of the study—e.g., numbers potentially eligible, examined for eligibility, confirmed eligible, included in the study, completing follow-up, and analysed; Consider use of a flow diagram. | Results, “Patient Characteristics”; Figure 1 |
| 13b. Participants – non-participation | Give reasons for non-participation at each stage. | Results, “Treatment Continuation and Discontinuation” |
| 13c. Participants – flow diagram | Consider use of a flow diagram. | Figure 1 (flow/continuation overview) |
| 14a. Descriptive data – participant characteristics | Give characteristics of study participants (e.g., demographic, clinical, social) and information on exposures and potential confounders. Present the information in a table. | Table 1 |
| 14b. Descriptive data – missing data | Indicate the number of participants with missing data for each variable of interest. | Results — Figure 1 and figure/table captions (denominators vary by time point); Methods — Statistical analysis and handling of bias (multiple imputation for missing outcomes). |
| 14c. Descriptive data – follow-up time | **Cohort study:** Summarise follow-up time—e.g., average and total amount. | Methods — Outcomes and follow‑up; Results — “Treatment continuation and discontinuation” and Figure 1 (follow‑up up to 36 months; continuation/discontinuation summarized). |
| 15. Outcome data | **Cohort study:** Report numbers of outcome events or summary measures over time. **Case-control study:** Report numbers in each exposure category, or summary measures of exposure. **Cross-sectional study:** Report numbers of outcome events or summary measures. | Table 2; Figures 2–3 |
| 16a. Main results | Give unadjusted estimates and, if applicable, confounder-adjusted estimates and their precision (e.g., 95% confidence intervals). Make clear which confounders were adjusted for and why they were included. | Results, paragraphs 3–5 |
| 16b. Main results – category boundaries | Report category boundaries when continuous variables were categorised. | Not applicable (continuous outcomes not categorized). |
| 16c. Main results – risk | If relevant, consider translating estimates of relative risk into absolute risk for a meaningful time period. | Not applicable (risk translation not applicable to endpoints). |
| 17. Other analyses | Report other analyses done—e.g., analyses of subgroups and interactions, and sensitivity analyses. | Results, subgroup analysis paragraph |
| **Discussion** |  |  |
| 18. Key results | Summarise key results with reference to study objectives. | Discussion, opening paragraph |
| 19. Limitations | Discuss limitations of the study, taking into account sources of potential bias or imprecision. Discuss both direction and magnitude of any potential bias. | Discussion, last paragraph |
| 20. Interpretation | Give a cautious overall interpretation considering objectives, limitations, multiplicity of analyses, results from similar studies, and other relevant evidence. | Discussion, entire section |
| 21. Generalisability | Discuss the generalisability (external validity) of the study results. | Discussion, last paragraph |
| **Other information** |  |  |
| 22. Funding | Give the source of funding and the role of the funders for the present study and, if applicable, for the original study on which the present article is based. | Funding — Manuscript, “Funding” section.  Competing interests — Manuscript, “Conflict of interest” section (verbatim text reproduced below). |
| Funding  No external or internal funding was received for this study.  Competing interests  HK reports lecture honoraria from Daiichi Sankyo Co., Ltd.; Eli Lilly Japan K.K.; and Otsuka Pharmaceutical Co., Ltd., outside the submitted work. The remaining authors declare that the research was conducted in the absence of any commercial or financial relationships that could be construed as a potential conflict of interest. | | |

**Supplementary Table S2.**

Treatment Persistence Over 36 Months: Numbers at Risk and New Discontinuations by Reason at Scheduled Assessments

| **Time point** | Baseline | 1 month | 3 months | 6 months | 1 year | 3 years |
| --- | --- | --- | --- | --- | --- | --- |
| Number at risk (on therapy at visit, n) | 50 | 48 | 48 | 42 | 36 | 28 |
| Evaluable for outcomes at visit (n) | 50 | 48 | 48 | 42 | 36 | 28 |
| New discontinuations since previous visit: Treatment completion (n) | - | 0 | 0 | 1 | 3 | 8 |
| New discontinuations since previous visit: Self-discontinuation (n) | - | 2 | 0 | 2 | 2 | 0 |
| New discontinuations since previous visit: Referral (n) | - | 0 | 0 | 3 | 0 | 0 |
| New discontinuations since previous visit: Switch to another CGRP agent (n) | - | 0 | 0 | 0 | 1 | 0 |
| New discontinuations since previous visit: Adverse event-related (n) | - | 0 | 0 | 0 | 0 | 0 |
| Continued on therapy at next scheduled visit (n) | - | 48 | 48 | 42 | 36 | - |
| Cumulative discontinued (n) | - | 2 | 2 | 8 | 14 | 22 |

**Supplementary Table S3. Sensitivity analyses for change from baseline at 1, 3, 6, 12, and 36 months (MMD, HIT‑6): model‑based time‑point mean changes (GLMM) and multiple imputation (m = 50)**

Notes. GLMM specification: Gaussian distribution, identity link; random intercept for patient; Time as categorical fixed effect (baseline value and EM vs HFEM/CM were optionally included where applicable). Model‑based time‑point mean changes (estimated marginal means) and 95% confidence intervals were derived from the fitted GLMM. p‑values are two‑sided Wald tests.

Multiple imputation: m = 50; change‑from‑baseline imputed at each time point; estimates combined with Rubin’s rules.

| **Outcome (MMD)** | 1 month | 3 months | 6 months | 1 year | 3 years |
| --- | --- | --- | --- | --- | --- |
| Estimated change (LSMean) | −5.761 | −6.710 | −6.756 | −6.908 | −6.519 |
| 95% CI (lower) | −9.49 | −10.44 | −10.55 | −10.75 | −8.83 |
| 95% CI (upper) | −2.03 | −2.98 | −2.97 | −3.07 | −4.21 |
| p-value† | 0.003 | 0.001 | 0.001 | <0.001 | <0.001 |

† p‑values are Wald tests from the fitted GLMM (two‑sided).

| **Outcome (HIT-6)** | 1 month | 3 months | 6 months | 1 year | 3 years |
| --- | --- | --- | --- | --- | --- |
| Estimated change (LSMean) | −10.854 | −9.250 | −10.452 | −11.515 | −14.037 |
| 95% CI (lower) | −15.45 | −13.95 | −15.12 | −16.12 | −17.21 |
| 95% CI (upper) | −6.26 | −4.55 | −5.78 | −6.91 | −10.86 |
| p-value† | 0.016 | 0.018 | 0.084 | 0.247 | <0.001 |

† p‑values are Wald tests from the fitted GLMM (two‑sided).

**Supplementary Table S4. Non‑serious adverse events during anti‑CGRP mAb treatment (safety population, n=50)**

| **Adverse event (AE)** | Patients with ≥1 AE, n (%) | Serious adverse event | Typical timing / course | Management | Led to discontinuation |
| --- | --- | --- | --- | --- | --- |
| Injection‑site pain | 5 (10.0) | No | Recurrent (approximately at each injection); pain complaints diminished over time in 3/5 patients | None / observation | No |
| Injection‑site erythema | 8 (16.0) | No | After first injection in all affected patients | Symptomatic treatment in 6/8; none in 2/8 | No |
| Constipation | 0 (0.0) | - | - | - | - |
| Hypertension | 0 (0.0) | - | - | - | - |
| Any AE (patient-level) | 10 (20.0) | - | - | - | No |

**AEs were identified by retrospective chart review during treatment exposure (from initiation to discontinuation).**

**Counts represent the number of patients who experienced ≥1 occurrence of each AE (patient‑level counts), not the number of episodes.**

**Three patients experienced both injection‑site pain and erythema; therefore, the sum of event‑specific counts (5+8) exceeds the number of patients with any AE (10).**

**All AEs were non‑serious; no serious adverse events (SAEs) were documented.**

**No patient discontinued treatment due to AEs.**
